# Navigating the infodemic: A qualitative study of university students’ information strategies during the COVID-19 pandemic

**DOI:** 10.1177/20552076241228695

**Published:** 2024-01-30

**Authors:** Lieve Gies, Mayuri Gogoi, Christopher D. Bayliss, Manish Pareek, Adam Webb

**Affiliations:** 1 Department of Media and Communication, 4488 University of Leicester, Leicester, UK; 2 Department of Respiratory Sciences, 4488 University of Leicester, Leicester, UK; 3 Department of Genetics and Genome Biology, 4488 University of Leicester, Leicester, UK; 4 Development Centre for Population Health, 4488 University of Leicester, Leicester, UK; 5 NIHR Leicester Biomedical Research Centre, University Hospitals of Leicester NHS Trust, Leicester, UK

**Keywords:** Misinformation, infodemic, COVID-19, media literacy, experience, pandemic, vaccine hesitancy, media use, social media

## Abstract

**Objectives:**

We aimed to study the strategies which university students developed for vetting information during the COVID-19 pandemic and associated infodemic.

**Methods:**

We conducted semi-structured interviews with 34 students, using a piloted topic guide which explored several areas of pandemic experiences, including students’ use of media. Transcripts were analysed inductively following the thematic approach. Higher order themes were finalised following a coding exercise undertaken by two of the authors.

**Results:**

Participants were acutely aware of misinformation during the pandemic. They rated legacy news media (print and broadcast media with pre-Internet origins) higher than social media for reliable information about the pandemic. However, strikingly, not all legacy media were automatically trusted and not all social media were uniformly distrusted. Participants identified a set of mechanisms for establishing whether a piece of information was truthful and accurate. These mechanisms had four main focal points: (1) the source, (2) the message, (3) individual media literacy and (4) the trustworthiness of others. Despite possessing a critical awareness of misinformation, participants avoided posting anything in relation to the pandemic for fear of becoming the target of online abuse.

**Conclusions:**

In addition to underscoring the role of media literacy, our research foregrounds the need to attend to the importance of fostering media confidence. We define media confidence as the ability of digital media users to challenge and interrogate questionable or inaccurate information safe in the knowledge that there are adequate regulatory mechanisms in place to curb abuse, trolling and intimidation.

## Introduction

At the beginning of the COVID-19 pandemic, Tedros Adhanom Ghebreyesus, Director-General of the World Health Organisation (WHO), famously declared that ‘we are not just fighting a pandemic; we are fighting an infodemic’.^1^ While misinformation and conspiracy theories are not new phenomena,^2^ it was the scale of misleading information and the ease with which it was able to spread that stood out during the pandemic. The concept of an ‘infodemic’ challenges the notion that the digital media user is empowered by the knowledge available online: instead, it envisages a user overwhelmed and disoriented by a deluge of information, much of which is of questionable value. The repercussions for public health are potentially very significant as the information environment may be a key determinant of how well a viral outbreak is contained.^3^

Building the necessary resilience to misinformation is a key strategy in mitigating the impact of an infodemic by equipping communities and individuals with the skills and knowledge required to distinguish between trustworthy and untrustworthy information. In this article, we present the findings from a study in which university students were interviewed about their strategies for vetting information in the middle of the national COVID-19 lockdown in the United Kingdom in the summer of 2021. At the time, university students were of particular interest as a population as they typically live in shared accommodation and are geographically very mobile, making them a key target for a range of preventative public health measures aimed at containing the highly contagious COVID-19 virus. A central research question for this study was the way in which students went about identifying misinformation. At the time, digital interfaces afforded not just a window on the world, but, owing to social distancing rules, they were also the main gateway to a whole range of social activities, including working, learning, shopping, engaging in leisure activities and keeping in touch with family and friends, making the online world an indispensable part of everyday life. This meant that staying offline was not an option for many people and that, consequently, misinformation was something that needed to be confronted by individuals on an almost daily basis. Against this backdrop, we wanted to explore students’ awareness of the ongoing infodemic and the strategies which they deployed to filter out misinformation. For our method, we opted for semi-structured interviews as these enabled participants to give a detailed account of their lived experiences, with plenty of scope for interviewers to ask follow-up questions as required.

In what follows, we first offer an overview of some of the principal research literature on misinformation during the COVID-19 pandemic. Secondly, we set out our research design. Thirdly, in our presentation of our findings, we provide a general overview of the information sources which interview participants found the most and the least trustworthy, along with a presentation of findings of how they accessed information and used social media platforms during lockdown. We will also detail how participants described the mechanisms which they deployed for distinguishing between trustworthy and untrustworthy information. Fourthly, in our discussion, we explore the implications of our findings for tackling health-related misinformation, including the need to not just equip users with the skills to identify trustworthy information and resist misinformation, but also to create a media ecology which gives individuals the confidence to question and challenge misinformation.

## Literature review

Research into media use during the COVID pandemic has highlighted the distinct role which social media have played in enabling misinformation to circulate. Findings do not only suggest that there was heavy reliance on social media during the pandemic, but also that many users were unlikely to fact-check online information, despite displaying high levels of distrust of such sources.^4^ Song et al.^5^ show how misinformation about COVID-19 was shared more frequently on social media than was accurate information. However, a key finding from the study by Cinelli et al.^3^ is that there were no significant differences in the spread of unreliable and reliable information online. Analysing COVID-19 misinformation content in TikTok videos, Baghdadi et al.^6^ found that videos with a high grade of misinformation were less likely to attract views but, at the same time, these were more likely to generate active user engagement, for example, in the form of comments expressing an intention to change behaviour in response to the misleading videos. On a more positive note, Kim et al.^7^ in a secondary data analysis of a large-scale survey in the United States found that social media information, along with interpersonal communication, was the most effective at translating knowledge about COVID-19 into the adoption of protective behaviours.

The proliferation on COVID-19 misinformation on social media has prompted researchers to evaluate the importance of media literacy and associated skills in mitigating the impact of misleading information. For example, the possession of media literacy can lead to a greater willingness to adopt recommended protective measures against COVID-19 infection. Austin et al.^8^ note that that ‘skillful navigation of the information environment (…), including attention to expert sources can facilitate more effective analysis of a changing information base’ (p. 248). The latter factor was especially important in the context of ever-evolving and contradictory information about COVID-19. In a large international study involving multiple countries, Roozenbeek et al.^9^ found that, notwithstanding the finding that public belief in COVID-19 misinformation was not widespread, misinformation still managed to attract a significant proportion of believers. Conversely, a minority struggled to believe trustworthy information about COVID-19 vaccination. These findings also support the notion that a belief in misinformation negatively impacted the intention to be vaccinated and to adhere to public health guidance. Higher numeracy skills and higher trust in scientists were associated with a reduced susceptibility to COVID-19 misinformation.^10^ Ashley et al.^11^ found that news media literacy can be effective in reducing susceptibility to COVID-19 misinformation; however, this is predominantly the case for individuals holding liberal political viewpoints. They also note the uneven distribution of media literacy across different parts of the population. A related strategy for coping with misleading health information is cognitive reflectiveness, defined by Weil and Wolfe^10^ as ‘an individual's ability to avoid incorrect intuitive responses by being more thoughtful and reflective in their judgment and reasoning process’ (p. 20). Choudrie^12^ used interviews to examine how older adults (aged 50 and above) processed online misinformation about the pandemic. They found that research participants trusted information about COVID-19 from legacy media (print and broadcast media with pre-Internet origins) more than online content because they were confused about the veracity and authenticity of online information. While disregarding online media in favour of legacy media may be a useful strategy for avoiding misinformation, Su et al.^13^ sound a note of caution, pointing out that, despite the higher journalistic standards of legacy media, some news outlets featured baseless and sensationalist stories in their reporting of the pandemic.

As already noted, trust in scientists represents another relevant protection against COVID-19 misinformation. Stjernsward et al.^14^ report that health authorities, health experts, WHO and other scientists were especially trusted sources for information about COVID-19 in Denmark and Sweden. Hernandez et al.^15^ introduce the concept of ‘health care provider social media hesitancy’ to describe and problematise the reluctance of health professionals to provide factually correct information about COVID-19 vaccines on social media, pointing to the dominance of anti-vaxx discourses. Prasad,^16^ analysing a conspiracy video, shows how misinformation about COVID-19 misappropriated the credibility of scientists, science and scientific journals. These findings suggest that lay audiences may find it challenging to distinguish between false and accurate scientific claims.

One of the most intriguing aspects of the pandemic was its politicisation and the influence of political orientation on, for example, a person's belief in the seriousness of the pandemic and their willingness to be vaccinated. Motta et al.^17^ observe how the right-wing media in the United States were the main sources to spread misinformation about COVID-19 in the early stages of the pandemic. Their findings also suggest that individuals’ consumption of right-wing media was related to misinformed views about COVID-19. Olivett et al.^18^ conducted a quantitative study among undergraduate students in the United States, demonstrating a link between conservative political beliefs and lower engagement in public health behaviours during COVID-19. However, they also established that this relationship was mediated by the ability to identify misinformation and by the perceived threat individuals associated with COVID-19. Using a large-scale survey of US population, Sylvester^19^ found a link between ideology and levels of education, on the one hand, and knowledge of COVID-19, on the other. Liberal respondents overall answered questions about COVID-19 more accurately. Reisdorf et al.^20^ identified gender, income levels and levels of education as important factors determining how many information sources an individual will use, with men, high earners and the highly educated showing a propensity to use a wider range of sources, leading to better fact-checking of online information about the pandemic. Respondents who were younger, had left-leaning political views and reported possessing above average online navigation skills also tended to engage in more extensive fact-checking.

Indeed, education, along with political orientation, is frequently singled out as a relevant variable in the literature on COVID-19 misinformation. Delmastro and Paciello^21^ observe in a cross-sectional study of the Italian population that individuals who were most at risk of believing in misinformation tended to have higher rates of depression and lower levels of education. Filkukova et al.^22^ conclude from their Norwegian survey that being male, having lower levels of education, decreased consumption of news media and increased Internet use, along with high levels of trust in social media information, generally predisposed individuals to endorse COVID-19 misinformation. They also note that both COVID-19 scepticism and the belief that the threat from COVID-19 was overstated were risk factors for endorsing misinformation.

The effects of misinformation include vaccine refusal and non-adherence to preventative health behaviours. Lockyer et al.^23^ report the findings of a small qualitative study involving telephone interviews with people in Bradford (UK) in which the researchers aimed to understand participants’ beliefs about COVID-19 against the backdrop of low vaccine uptake in the city. Their findings suggest that misinformation fuelled confusion, distress and mistrust. The greater these effects, the less likely participants were to take up COVID-19 vaccination. However, in a follow-up study, the researchers re-interviewed 12 of the 20 original participants and found that 11 out of 12 had managed to overcome their concerns about the vaccine.^24^ Comparing different types of media, Allington et al.^25^ found a positive correlation between the intention to be vaccinated against COVID-19 (bearing in mind that at the time of the study a vaccine had not yet been approved) and the use of legacy media. Furthermore, for respondents relying on social media for their information, the study reported a negative impact on the intention to be vaccinated. However, the findings also suggest that frequency of social media use in itself did not have any significant effect. Similarly, Jennings et al.^26^ found a potential relationship between the use of YouTube and increased vaccine hesitancy in respect of COVID-19, suggesting that the prevalence of videos featuring negative representations of vaccines on the platform provided a potential explanation. In a survey conducted in Serbia, Teovanonic et al.^27^ found that irrational beliefs (especially beliefs in conspiracy theories) were predictors of an unwillingness to get vaccinated and adhere to public health guidelines.

The existing literature has documented the complexities of users’ consumption of information during the pandemic and their susceptibility to COVID-19 misinformation. The variables that determine whether individuals are drawn to misinformation are manifold: demographics, choice of platforms and information sources, but also the way and extent to which users fact-check and actively assess the trustworthiness of the available information are all relevant in understanding the differential impact of the COVID-19 infodemic. The majority of studies cited in this section have used large-scale surveys which have been able to yield statistically meaningful data about the multi-factorial dynamics of COVID-19 misinformation. However, there is further scope for understanding the way in which individuals evaluate the mechanisms which they deploy to resist misinformation. Such reflectiveness is instrumental in providing media users with the impetus not just to be vigilant to the risks of misinformation but also to actively seek to bolster their media literacy skills. In what follows, we will draw on reflective accounts obtained through in-depth interviews with undergraduate university students to provide insights into strategies which participants described to cope with misinformation during the pandemic.

## Methods

### Theoretical framework

A key tenet of audience studies is that media audiences do not passively absorb the message embedded in media texts; instead, they have the capacity to fashion their own meanings.^28^ Stuart Hall's^29^ pioneering insight that the ‘decoded’ message may vary greatly from the ‘encoded message’ accorded audiences the capacity to actively resist ‘preferred’ meanings. Methodologically speaking, this novel approach coincided with a shift towards ethnographic and other qualitative research methods to capture audiences’ creativity and resistance. In what has been termed the ‘post-audience age’,^30^ digital media have caused the very boundary between media and audience to be blurred. In Deuze's^31^ words, we no longer ‘live *with*, but *in* media’ (p. xiii), resulting in the concept of the ‘audience’ to be replaced with that of the ‘user’ or even ‘produser’ to reflect the high levels of active media participation and content creation in a society in which so many human activities require interaction with a screen. The COVID-19 pandemic was the epitome of the digital revolution with large swathes of the population turning to Zoom and other virtual meeting platforms to enable everyday life, such as work, education and even medical care, to carry on amidst mandatory lockdowns and social distancing. Yet, when the WHO declared an ‘infodemic’ alongside the pandemic, the notion that misinformation could be as harmful and contagious as the novel Coronavirus itself to a large extent envisioned digitally savvy users as passive and helpless against the onslaught of misinformation. For media scholars, such thinking aligns with the old ‘hypodermic syringe’ model of direct media effects and ‘passive’ audiences in which the central question is ‘what media does to people’ rather than ‘what people do with the media’^32^ (p. 2). Misinformation presents something of a paradox in the digital era in which media consumers have unprecedented access to knowledge and thus possess a considerable degree of agency, but are simultaneously disempowered because they are struggling to recognise what is genuine, bona fide information in an endless and fastmoving supply of information. Our research design is premised on the idea that such incongruities reflect the realities of contemporary digital life: consequently, it considers the data from the lens of both the ‘active’ and ‘passive’ audience models. A key focus of our study was to explore in depth the ways in which research participants grappled with the challenges of misinformation at the height of the COVID-19 pandemic and the mechanisms they used for navigating the infodemic.

### Study setting, participants and recruitment

The study was conducted in a public funded university in England that has an average enrolment of 19,000 students annually. The university has an ethnically diverse student population. The qualitative study was part of a larger mixed-method study on students’ pandemic experiences including their attitudes, knowledge and intentions for uptake of COVID-19, MMR (Measles Mumps Rubella) and MenACWY (Meningitis) vaccines.^33^ The study population included undergraduate students enrolled in any course at the University. Undergraduate students were selected because the COVID-19 vaccine was only just made available to all 18-year-olds in the United Kingdom at the time and we therefore assumed that most students would be unvaccinated when they completed the questionnaire.^33^ The survey was completed by 827 students pursuing a range of courses across different disciplines. One hundred and eighty-seven of these students expressed an interest to take part in the qualitative interviews and they were contacted through the email provided, with an invitation and further information about the qualitative study.

### Data collection

Out of 187 who expressed an interest, finally 34 students took part in the qualitative interviews. Relevant socio-demographic details are provided in Table 1. Interviews were conducted via Microsoft Teams between July and September 2021. Demographic details of the participants were retrieved from their survey responses. LG and MG conducted the interviews using a piloted topic guide which explored several areas of pandemic experiences including students’ use of media during this time. Both LG and MG are trained qualitative researchers who have experience of conducting research with different population groups, including students and ethnic minorities. LG is an associate professor at the University and we realised that this could have an influence on the responses of students’, particularly those attending her course. She therefore consciously decided not to interview participants from her course and these participants were interviewed by MG instead. Interviews lasted between 30 and 90 min and were recorded with prior permission using the recording feature in Teams. Recorded interviews were transcribed verbatim using transcription software and a professional transcriber. All transcripts were anonymised; in the excerpts below, we have chosen to identify participants by a randomly allocated number.

**Table 1.** Characteristics of interview participants.

Participants’ characteristics	N (%)
Sex	
Female	21 (61.7)
Male	13 (38.3)
Ethnicity	
White (including non-British White)	19 (55.9)
Asian	10 (29.4)
Black	2 (5.9)
Other	3 (8.8)
Age	
≤ 22	20 (58.8)
22+	14 (41.2)
Year of study	
First (including foundation)	13 (38.3)
Second	14 (41.2)
Third	7 (20.5)
Course	
Humanities	12 (35.2)
Law	4 (11.8)
Life science	8 (23.5)
Medicine & allied	4 (11.8)
Natural science	4 (11.8)
Social science	2 (5.9)
Residence status	
UK student	26 (76.4)
UK-based international students	4 (11.8)
Non-UK based international students	4 (11.8)

### Data analysis

A thematic analysis approach^34^ was applied and transcripts were analysed by LG and MG wherein they first let the data drive the coding process and once a coding framework was established used it to code subsequent transcripts. More specifically, firstly, the two authors (LG and MG) read a few transcripts each to familiarise themselves with the data. Thereafter, they independently coded these transcripts on NVivo and discussed the coding framework at regular progress meetings. The field notes, made after every interview, were also used to guide the discussions at these meetings. This enabled them to arrive at a working coding framework which was then used by the two authors to jointly code three additional transcripts. This exercise led to some further modifications to the coding framework. The emergent framework was then used by the two authors to independently code the remaining transcripts. Finally, when all the transcripts had been coded and data saturation had been agreed, the authors had discussions to interpret, rearrange and identify connections among codes to arrive at the themes and sub-themes which are reported in the paper as per the Consolidated Criteria for Reporting Qualitative Research (COREQ) guidelines.

## Results

### Media and information uses

In this section, we offer a thematic overview of the patterns of media and information use which interview participants described. We focus on the sub-themes of (a) trusted sources of information and (b) modes of media access and social media use.

#### Trusted information sources

When it comes to trust in various information sources, our interview findings suggest that the choices which participants made were more subtle than a simple trade-off between legacy media and social media. A good illustration is the following interview excerpt:

OK, *Daily Mail* – mistrust! There was like obviously I still look at it because I just want to know what they're saying. WhatsApp videos – mistrust. I don't believe in them. I've watched some of them and I've just thought that they're so absurd. What else…most online stuff, like, there's not – very rarely that you're going to 100% believe in it. (P32)

Many participants expressed a preference for broadcast news media with a reputation for balance and impartiality over what were, in their views, biased news channels such as Fox News. Similarly, broadsheets were preferred over tabloid newspapers, and while ‘left-leaning’ media were not mentioned explicitly as being especially trustworthy, it was implied by the perception of right-leaning media organisations as especially untrustworthy. Other trusted sources included scientists (e.g. via peer-reviewed papers), who were rated more highly than were government and individual politicians, as is evident from these excerpts:

I think if it comes from reputable sources such as official news channels and also medical journals and all medical websites such as NHS or any other established website that uses, consults qualified experts or fact checks information, I think information from them is trustworthy and credible, but then there's a lot of online, there's a lot of fearmongering and bandwagon-ing misinformation and all of that. (P17)

I don't trust things directly from the government because it is the government and I don't particularly like the current party that are in power. And so I feel like there's always something hidden when they say something. (P05)

As far as the Internet and social media are concerned, the picture is equally nuanced: while social media were described as the worst sources of COVID misinformation, there were some subtle differences. Twitter (nowadays known as ‘X’) is an interesting example. Verified accounts linked to scientific bodies and established news organisations were named by some participants as being among the most trustworthy. Furthermore, although Twitter was not universally rated as trustworthy, some participants trusted Twitter more than Facebook, as is evident from the following interview excerpts:

I think I trust Twitter more than Facebook, because for some reason for Facebook you can easily see fake news, because on Twitter the most relevant tend to be the ones with most interactions so like most ‘likes’ or ‘retweets’, where on Facebook even random posts that are from fake sources can appear on your timeline and I see my auntie retweeted it even though it's from a dodgy website or something and she's believing that oh this vaccine caused this side effect. But on Twitter, those ones from dodgy websites don't get much interactions so they won't appear on your main page or main timeline, so I prefer it to most really. (P02)

I think unless it's from an actual news source, Facebook I just think that's false until proven true because of the way that it is. And the same with most things on social media, I think. I will read it until I've proven it true. I also find with *Daily Mail* and *Daily Telegraph*, everything they say is taken with a big cup of salt! (P10)

However, as already observed, Twitter was not seen as trustworthy across the board:

[I don’t trust] anything anyone says on Twitter. I just don’t, I just accept that people are going to make up stuff and whatnot so that, all social media opinions are just that. I don’t really take them seriously. (P13)

I like Twitter for some things (…) don't get me wrong, (…) Twitter probably has its fair share of (…) misinformation. (P06)

TikTok, the social media platform that very much came into its own during the pandemic and is particularly popular among younger age groups, was not referenced all that frequently in terms of its trustworthiness. Nevertheless, it attracted some contrasting assessments:

Yeah, definitely [I trust TikTok], because I feel like it's because a lot of the demographic is people my own age. And I feel like I can trust people my own age rather than people who are older than me and maybe not experience the same things that I have essentially. (P05)

I don’t use TikTok either. I just think it's too much false information. (P27)

#### Modes of access and social media use

As for modes of access to information about COVID, it was striking, but not surprising, that our young cohort overwhelmingly accessed news and information online. The concept of watching a broadcast on a TV set or reading a paper copy of a newspaper was almost completely alien to participants. Instead, participants principally used their smartphones to access news sources. Streaming of both live and recorded events was the dominant way of following important news events about the pandemic (e.g. daily government press conference at the height of the pandemic):

**Participant:** I go to multiple news outlets so that the BBC, Sky News, newspapers such as *The Telegraph*, multiple sources.

**Interviewer:** OK, and do you access these online, on the TV or physical copies of newspapers?

**Participant:** Primarily online? I would say almost entirely online. (P03)

Many interview participants made a clear distinction between using social media to access information and for non-information purposes, such as socialising, entertainment and hobbies:

[I] don't really get much [news] through Facebook about COVID, to be honest, but I use Facebook in a different way. I wish [I wasn’t on social media]! I am because – again, this is a whole other topic – but I kind of don’t think it's good for mental health in general to be on it, but the kind of social aspect of it kind of keeps me on it, being able to chat to friends and being able to socialise with friends. But in general I don’t think it's very good for you. (P18)

Weirdly, I have a Facebook page that I don’t really use, it's just there to, if I wanted to find an old friend, it would be there to go to and exchange phone numbers rather than talk on there. (P24)

I probably go on [YouTube] about once a day. Well, a few times a day. I use it as one of my primary sources of entertainment I’d say. (P04)

One participant mentioned peer pressure to get a TikTok account in order to keep in touch with others:

Because I just didn’t want a Tik Tok account but I got peer pressured into it, I got it recently. But I don't think I use Tik Tok for news though. Definitely there are a lot of educational people actually on Tik Tok, like older scientists who use it now and people who use it to like contact the ‘youth’ and things like that. (P32)

A clear sign of a passive engagement with COVID-19 information is that most interview participants never or very rarely posted anything in relation to the pandemic for fear of becoming the target of trolling, abuse and negative comments. Despite their awareness of the online information environment (and indeed because of this awareness), they would not challenge any misinformation they encountered, or, alternatively, endorse any accurate information. COVID-19 vaccination provides an insightful example: research participants were mostly very supportive of the vaccine, but no one we interviewed felt confident enough to discuss their vaccination status openly online. Two participants cautiously signalled their support for the vaccine by posting a photo of their COVID-19 vaccination card on their Instagram page but without any further explanation:

I don't really tend to start of any controversial discussion on social media in general because it can be quite divisive conversations. A lot of people are posting on Instagram, so like showing your vaccination card. And I have done that. So, I kind of have endorsed it [vaccination], but I haven't started a conversation about it. So [the photo] was like basically saying that, like, I'm getting [vaccinated]. (P06)

I don't really to get involved in that many online discussions because they tend to like… somehow someone twists it into an argument and I can't really be bothered with that kind of thing. I think when you're talking about something in person, it doesn’t really evolve in that same way, whereas a lot of the time online it seems to find its way to being an argument even if people are kind of on the same side, which is fine, I don't mind, some people like getting into that kind of discussion, but it's not really for me, so I just tend to avoid that kind of thing. (…) I don't really post much stuff to do with [COVID]. I’ll just mention in the caption [on Instagram] like when I got my vaccine I was like: yeah, [I’ll] mention that because I'm quite happy about that, but [the post] wasn’t centred around that. (P30)

### Misinformation vetting methods

Our next theme details the strategies which participants outlined for trusting specific sources and the reflective awareness they demonstrated in describing these methods. Participants identified a set of mechanisms for establishing whether a piece of information was truthful and accurate. These vetting mechanisms had four main focal points: (1) the source, (2) the message, (3) individual media literacy and (4) the trustworthiness of other people. These four categories are not mutually exclusive; on the contrary, interview participants often described using a combination of strategies in establishing the trustworthiness of COVID-19 information.

#### Scrutinising the source

Participants made several references to the governance of information sources. One example is the presence of community standards on online platforms:

[I prefer Reddit] because it's not toxic, it's not a toxic place because it's not Twitter, it's just likeminded people and there are rules to each community so like if you swear, that kind of stuff, it's very like no it won't get posted then, they’ve got a lot of monitoring system where like they don't want that kind of energy there it's not going to come on and the community don't see it then, might have to rework the whole thing if they even wanted to write anything. (P01)

A related aspect is the reassurance offered by media regulation and strong editorial practices attributed to established news media. An extension of such practices is adherence to journalistic independence and impartiality. For example:

I trust any regulated news, you know, one that doesn't engage in gossip columns this way and it's sort of, I won’t say legitimate, but any sort of newspaper whose focus is on strong editorial practices and presenting an argument. (P03)

So a lot of the time it was (…) the BBC [I trusted]. I know that those news outlets can’t be reached by political pursuits or monetary pursuits and things like that. (P28)

Assessing whether a source possesses the appropriate qualifications and authority to make a particular statement is another form of source scrutiny. This especially applied to non-journalistic sources, including scientists, medical experts and official, institutional sources such as the NHS, PHE (Public Health England) and the WHO:

When I think it's like when like the World Health Organization or like Public Health England picks up that thing [of Astrazenica vaccine carrying a risk of blood cloths], then you are more like if it's like a reliable organization, it's like picking up and like endorsing the move to like, remove [the vaccine]. Then you're a bit more, OK, that it's a scientific move. (P06)

To be honest, if I'm really, like, not entirely sure if it's true or not I’ll just look it up myself, cross reference different articles, different things, see what I believe, see who said what. Often, I’ll go to government websites or NHS [National Health Service] websites and see what they say (P20).

I use like the WHO website, the government website much more than I would use any other news website because I know that some journalists can sometimes get things a little mixed up and that's fine. Their job isn’t to be a doctor you know. I’d rather hear it straight from the doctors’ typing or mouth. (P28)

However, this does not mean that scientific credentials were always trusted, as these participants explained:

I’ve got some strong opinions on the World Health Organisation and their characterisation of the pandemic at times, so certain places that should be trustworthy I don’t find trustworthy for sure. (P04)

So I follow a few epidemiologists from Public Health England, so I know what they're tweeting is of course factual, but if it's someone like independent SAGE [Scientific Advisory Group for Emergencies] it gets utterly disregarded in the first instance, Independent SAGE [consisting of scientists independent of government], I do not believe a single word they say, but if it's going on government SAGE I don't believe everything but I know it's got the scientific backing or actual science behind it, not Independent SAGE, so I know, yeah, I try to always stay [with] accurate peer reviewed sources. (P15)

It is clear that reputation was a key factor enabling participants to trust information implicitly. Established news media and well-known institutions were regarded as purveyors of truthful information on the basis of their previous track record:

**Interviewer:** You've already said that you trust the the big news outlets like CNN, BBC. Are those the ones you trust the most for information?

**Participant:** I think, because they've proven they over a very long period of time, that they provide accurate information, I think. I think so, yeah. Yeah.

[I trust] high-profile sites like *The New York Times*, which has a history, which has a really like, they have a lot of people working for them and they have a reputation which they need to secure and so on. (P09)

At the same time, the interviews provide evidence that a good reputation can become tarnished or diminished. In the following excerpt, the participant reflects how over time their trust in Professor Chris Whitty, the Chief Medical Officer for England, had somewhat declined:

Like often I listen to, you know, the standard scientist that we have, Chris Whitty, but I felt that as the pandemic has gone on. Not that he's not reliable, but I think because he's tied to government, people are a bit more (…) like is he just saying what they're telling him to say really? I don't know. I think he still is a reliable source, but… (P06)

Participants also made a number of observations as to why they did *not* trust specific sources. These reasons included a perception that some media exaggerated aspects of the pandemic, the belief that misinformation was rife on specific platforms and the negative reputation attributed to the tabloid press, as well as a perception that some news outlets displayed a right-wing bias. For example:

I feel like a lot of news organisations, specifically like tabloid journalists and newspapers, most TV news networks, have made the pandemic seem so much worse so that people stay home and just keep watching and watching and watching. (P04)

To be honest I almost want to say I detest the social media and people that are getting their news from it. There's so much misinformation going around and I’ve got people, friends, who are kind of anti-lockdown, anti-vaccine, who are constantly kind of trying to share stuff to me and my friends that they’ve found online and it's just rubbish a lot of it. I mean obviously there's basis to some of it but it's just a lot misinformation. (P18)

I don’t particularly trust *The Sun*. I think that's been pretty much ingrained in me because I'm from the north, just over the water from Liverpool. (P07)

The latter participant's comments are particularly interesting as they refer to the historic distrust of *The Sun* newspaper in their home city of Liverpool dating back to the newspaper's reporting of the 1987 Hillsborough disaster which wrongly blamed Liverpool football supporters for the disaster. These events happened a long time before this student was born, suggesting that distrust in a particular media outlet can be handed down from one generation to another to affect something entirely unrelated such as a publication's reporting of the pandemic.

#### Scrutinising the message

A few participants stated that they would always adopt a dose of ‘healthy scepticism’ regardless of the identity of the information source:

To be fair, even from verified news sources I am not always sceptical but I still have a little bit of, you know, oh yeah, you have to have a healthy scepticism about everything, even trusted news sources because, you know, obviously media companies, they are [] at the end of the day. So I'm sceptical about everything online but just with verified news sources I'm a little bit less sceptical because they're more likely to be true. (P17)

I don't think I would ever 100% fully believe a certain source, like, I'm never going to fully believe every article the *Leicester Mercury* put out. (P27)

These comments hint at the importance of a second important strategy to guard against misinformation, which is to scrutinise the message itself. This strategy is often combined with due diligence regarding the source. By far the most frequently cited strategy was triangulation which involves cross-checking a piece information across different platforms and sources.

But what I do like to do is if [a newspaper] cited the academic paper, I like to read the paper myself, so I can see what is being said rather than – because sometimes you have non-specialists writing the papers, they’ve read the papers and they're writing the news and you can see where something has been lost in translation essentially, so I like to mix and match essentially. (P10)

I would probably look around a bit as well. Ideally if you’re reading – more than the BBC I’d probably trust more kind of anything in like the BMJ or published papers, things like that. But for the most part, yeah, I trust the BBC. (P20)

I think as long as you take in lots of different resources and you know what all the other people are saying, then from there you form it. So I never use one article alone or one service alone, it was like using a mix of other things, using your own education what you know about what vaccines are, then from there coming from it. Like the things that people were like ‘oh it stops you from having kids’ and you're like OK I know what vaccines, how they work, that just doesn’t make sense to me. (P32)

Other message-based scrutiny includes checking different sides of an argument, scrutinising the evidence presented in a piece of information and assessing the overall quality and presentation of an article or post:

But usually if somebody online is just saying something without any evidence backing them up or they're just opinion I don't put much stock on that anyway, I either ignore it or I’ll try to do more research myself. (P17)

I suppose I look at their profile, see what their job is and then if it's something like scientific I might look into it more. And then the actual post, if it's got loads of abbreviations, not like Covid-19 I mean like slang abbreviations, I wouldn’t particularly trust that, or [I wouldn’t trust a message with] loads of spelling mistakes. (P26)

I wouldn't be able to pinpoint like specific kind of sites and stuff, but I think you can kind of judge from things whether they're just trying to – big bold headlines – and they're trying to make it clickbait-ey and you can kind of judge and be like yeah that's just trying to come up with, I don't know, the clickbait-ey stuff that doesn’t have any substance and if you see something you're not sure about you're going to look at other sources and make sure that that is a valid thing that they're saying. (P30)

Common sense was also referenced by several participants who explained that the manifest absurdity of specific types of COVID-19 misinformation was sufficient to discredit such messages:

How could anyone believe this [misinformation]? Because it goes against what I know as someone who studied biology, anatomy, physiology, it just makes no sense at all. (P07)

Oh how did I know it was fake…um…I think listen first, whenever – about new issues surrounding COVID – I always hear it first from again news channels and stuff like that and if there's an article then I always read the article first before I look at other people's opinions, so if what people are saying clash with that and it's obviously stupid and it's an obvious example of misinformation, for example like the vaccine has microchips in it, you know, like some people were talking about before, that's definitely 100% I think know that's fake news and it's just fearmongering and it's just people that are ignorant. (P17)

Said a thread: ‘Why I think Satan is injecting something into my arm if I take the vaccine’. I was like, well. OK, so I read her thread and I was like, there's no logical sense. (P29)

#### Media literacy skills

A third factor which interview participants described as important in vetting misinformation is media literacy. These are the skills that are needed by media users to be able to scrutinise and challenge a piece of information. Here, trust is not determined by the face-value of the source or the message, but by an individual's ability to accurately judge the trustworthiness of information. While media literacy may require specific knowledge of the media, it also involves generic and transferable competencies, such as the aforementioned common sense. Thus, participants highlighted the importance of a specific understanding of how the media operate, as well as that of critical thinking, education and learning more generally in identifying problematic claims about COVID:

I try to use my critical thinking skills always as much as possible, though, as anybody else. I need to develop those kinds of things, especially especially this time when a lot of information are just going through your head and so on. (P09)

I just use my own judgement and I try to either just look at other sources to determine if it's real, or like sometimes it's just common-sense, like, I remember seeing videos of people sticking like a magnet on their arm and saying the vaccine has made them magnetic! But when you see things like that, some people might believe, but personally I just – you can't believe that because I just know from my education that it's not true, yeah. (P12)

You get that kind of confirmation bias and then the algorithms that social media uses, you’re engaged in that content and so it shows you more of that content and so you’re stuck in a constant echo chamber of hearing things that you want to hear (P20)

Being university students from different disciplines, participants evidently possessed varying types of knowledge. Thus, STEM subject students may apply a different body of knowledge to that of students in the humanities and social sciences. For example, this student references the paradigm of moral panics in their assessment of COVID-19 reporting which emanates from their specific academic discipline:

I mentioned I do Criminology as part of my degree and part of what we’ve been discussing in the past is things like moral panics, how the media can sort of inflame an issue to sell itself basically and that's what I feel like has been the case with the COVID. (P04)

#### Deferring to the judgement of trusted others

Fourthly and finally, in establishing what kind of information to trust, some interviewees stated that they were happy to defer to the critical judgement of trusted others. In this case, it is not the trustworthiness of the source, that of the information itself or confidence in their own media literacy skills, but trust in the ability of other people to make these judgements on their behalf that prevailed. In the observations made by our participants, family members figured more prominently than other groups, such as friends. This participant, for example, explains how they trusted their cousin to sift through the huge amount of COVID-19 information and identify trustworthy information, partly because they appeared to suffer from a lack of confidence in their own ability to spot misinformation, but also because of anxiety issues related to the pandemic:

And then a lot of it I get from my cousin because he's clued on on everything, he knows what's going on, so he’ll put it on our [WhatsApp] group chat and I’ll read that and then I'm like cool, that's enough information for me, that's all I need to know, because I trust my cousins a lot - I'm quite naïve and gullible so I’ll believe whatever you say kind of thing, so if it's someone I trust and it comes from them I'm like OK at least I know that that's something that's OK to believe in, because at first this whole lockdown situation had me so scared that I was losing sleep because of my anxiety from the whole situation, so I can't read it half the time because it really triggers me, so that's probably a reason why I don't keep up as well because I just get scared over things that don't even affect me most of the time! (P01)

Similarly, several participants described how they relied on information relayed to them by their parents, making the latter potentially important gatekeepers in the way in which information cascades down from media sources:

Yeah, to be honest, mainly in terms of, like, important updates I can't lie, my dad tells me most of them because he watches the news daily and he’ll be like ‘oh by the way this is happening, that's happening’. (P18)

And also my own parents because I know that they understand things like this better. My dad did a, he was training to be a doctor for four and a half years at university so he understands this a little bit more. (P27)

My pre-pandemic media source is my dad. My dad is the news junkie in the house. He's the one who always checks the news. I would say it's funny because before the pandemic, I'd probably check the news every once in a while, but I would mainly hear word of mouth through my dad, like say, oh, this is the news. I was like, wow. But funny story now that I've been home this past year, I sometimes go on the news myself. Like I'll just like look up the news myself. (P29)

The ability of trusted others to rectify misinformation was highlighted by this participant who discussed how one of their university teachers was able to put their mind at ease about the side effects of the COVID vaccine:

I will look a bit into it more, yeah, and try to find out is it true for example and if it is not and I’ll just leave it, if I don't know what it is, I will not either trust it – I will not be definite about it, yeah, or talk about it. I need to know exactly what it is, for example when they were talking about this Covid thing, they will put a nanochip in you and they will do this! And I was thinking how can it be like that – do you think it is true! And like that, I was just asking myself, when I was speaking to one of my lecturers, we just had like a little conversation, then she said ‘no, it is something to do with protein production and all that’, but still they don't know what is the side effects, that was a long time ago. Then when she told me that, then I started to make sense, OK so this is like this, the vaccine is being made like this, so yeah, and then you just all whatever the social media was saying, I knew it is wrong, they don't know exactly how these things are made, yeah, they're just saying it, I don't know, trying to make people being fearful about the vaccine. (P25)

These comments point to the importance of personal interaction to counteract misinformation and clear up any doubt or confusion that misinformation may have caused. Conversely, confirmation of inaccurate or fake information by trusted others may be capable of amplifying the effect of misinformation, although our interviews do not contain any immediate examples of this.

## Discussion

Our interview participants deployed several strategies for detecting and avoiding misinformation during the pandemic. While, in line with the research literature, legacy news media were rated more highly than were social media during the pandemic, the overall picture is nuanced in that not all legacy media were automatically trusted and not all social media were uniformly distrusted. The tabloid press is a case in point as a type of legacy media which generated considerable distrust among our interview participants. With social media, it was evident that, while collectively these platforms inspired limited trust, some – for example, verified Twitter accounts – were less distrusted than others. At the same time, with many activities taking place online during the pandemic and lives being lived *in* the media, our participants considered social media an unavoidable part of everyday life. They explained how they reluctantly used some of these platforms to connect with others and to find entertainment during lockdown, but also how they would consciously avoid or discount some social media as sources of COVID-19 information. Another type of avoidance behaviour is that participants very rarely posted anything about the pandemic as it was considered too risky a topic. Misinformation on social media was witnessed, but not actively challenged or corrected. Defined by Hernandez et al.^15^ as the reluctance of health professionals to spread factually reliable information about COVID-19 vaccination online for fear of reprisal, our findings suggest that the concept of social media hesitancy may also apply to other groups who, for similar reasons, were reluctant to openly share information online about the pandemic.

We noted earlier that the concept of an infodemic implies that the sheer volume of information during the pandemic overwhelmed and disempowered the active digital media user. Our findings indicate that our participants’ media use can be described as actively passive in the sense that they consciously and purposefully avoided, disregarded and limited their presence on specific media platforms, rendering themselves to a large extent invisible. At the same time, when we delve more deeply into the methods which interview participants used to identify and eliminate misinformation, we can characterise these strategies as a form of passively active media use. Keeping a low-profile online, participants appeared to be actively scanning the COVID-19 media landscape for misinformation. Potential red flags could come from both the source and the message, with vetting criteria including the extent to which a news source was regulated, its reputation and its qualifications, as well as message-oriented strategies such as triangulation and scrutiny of the overall quality of a message's presentation. Media literacy, encompassing a wide-ranging set of skills, provided an additional mechanism which participants deployed in evaluating the reliability of information. They also discussed the importance of trust in specific gatekeepers whom they regarded as reliable arbiters of misinformation. Participants’ reasoning in this regard very much fits the two-step flow of communication model^35^ which posits that media influence involves the intervention of opinion leaders who play a crucial role in passing on and endorsing media messages so as to give these messages resonance among a wider population.

Actively passive and passively active media use are defensive strategies. It appears that during the pandemic, participants chose to protect themselves from the infodemic through the digital equivalent of social distancing. Their engagement with social media was limited, selective and cautious. We know that misinformation about COVID-19 and other topics is often spread by a vocal minority.^6,36^ A very large number of users do not share nor give credence to conspiracy theories and other misinformation. Overcoming the hesitancy of a silent majority to speak out and provide a corrective to misinformation is arguably one of the greatest challenges posed by an infodemic. In addition to underscoring the role of media literacy, our research foregrounds the need to attend to the importance of fostering media confidence. We define media confidence as the ability of digital media users to challenge and interrogate questionable or inaccurate information safe in the knowledge that there are adequate regulatory mechanisms in place to curb abuse, trolling and intimidation. This would not just protect individual posters, but it would also act as a quality kitemark for platforms, especially as regards health information. While media literacy very much puts the onus on individuals, fostering media confidence is not just a matter of individual responsibility. If anything, it is something that requires collective action, making the creation of a safer digital media ecology a clear priority for public health.

## Conclusion

Our study suggests that research participants were acutely aware of the problem of misinformation during the pandemic. The choices which they made regarding trusted sources of information and modes of media use were highly nuanced, and not based on simplistic binary distinctions. Similarly, students were capable of articulating various strategies for detecting unreliable information that ranged from scrutinising the source to scrutinising the message. They were also influenced by their own media literacy as well as the trust in the judgement of significant others. Interestingly though, despite students’ awareness about prevailing media misinformation, they showed great reluctance when it came to openly challenging or correcting misinformation in online platforms. We described their use of media and information sources as both actively passive and passively active. We studied a population that is ethnically diverse and highly educated; results might vary in other population groups. The strength of our study is that its qualitative aspects allowed us to capture the complex challenges of navigating the infodemic at a point during the pandemic when there was still a great amount of uncertainty and public debate about the necessity and legitimacy of various public health measures, including vaccination.

Although this study has highlighted some very important aspects of university students’ media use during the COVID-19 pandemic, it is not without limitations, the most significant one being the low response rate. Although 187 survey participants expressed an interest to take part in the interviews, finally only 34 took part in the interviews. The relatively low participation rate could have been influenced by several factors, such as the ongoing exams during the data collection period, the time difference for international students who were not living in the United Kingdom at the time, and a general change in circumstances from the time of filling in the questionnaire to getting the interview invite. We actively tried to address this limitation by sending three reminders to all students who had expressed an interest but failed to respond to the invite. Nevertheless, for a qualitative study, the sample was sufficiently sizeable to generate data that is rich and nuanced, reflecting the complexity of behaviour and attitudes that surround media use. Another limitation of our study is that media use was self-reported by participants and not evidenced by studying actual online activity. Our study nevertheless affords insights into attitudes and perceptions that can have an influence on university students’ media use and these findings can be explored further in large-scale media analysis research. Larger studies involving different populations are needed to test and refine the various categories emerging from our research. Further research to examine the role and impact of misinformation following the pandemic would also help to put the acute nature of the infodemic into perspective by providing longitudinal data. Going forward, our findings support the case for an online environment which fosters users’ media confidence as an integral part of the battle against misinformation, especially but not exclusively during public health emergencies.
